# Analysis of the impact of traditional ethnic villages in Hani area on sustainable development

**DOI:** 10.1371/journal.pone.0283142

**Published:** 2023-03-16

**Authors:** Xinying Ma, Yu Shi, Shidong Zhang, Jingbiao Yang, Luo Guo

**Affiliations:** 1 College of Life and Environmental Sciences, Minzu University of China, Beijing, China; 2 State Key Lab of Urban and Regional Ecology, Research Center for Eco-environmental Sciences, Chinese Academy of Sciences, Beijing, China; Renmin University of China, CHINA

## Abstract

Rapid economic development and accelerated urbanization have seriously affected the development of traditional ethnic villages in China. We used the minimum cumulative resistance (MCR) model based on land use, landscape pattern, and ecosystem service value (ESV) to evaluate the spatio-temporal dynamics of sustainable development in Hani traditional ethnic villages from 1995 to 2020. By analyzing changes in sustainability indicators in the Hani area and different buffer zones, this paper aims to assess the impact of ethnic villages in the Hani Area on sustainable development and provide recommendations for the sustainable development of traditional ethnic Hani villages. The results indicated that: (1) The area of construction land and landscape fragmentation in the Hani area significantly increased and the value of ecosystem services and levels of sustainable development decreased each year during the study period; (2) The area of cropland in the 2 km buffer zone of the traditional ethnic villages increased, the degree of landscape fragmentation, the value of ecosystem services, and the level of sustainable development were lower than in the 4 km buffer zone during the study period. This is due to population increases in traditional ethnic Hani villages, as well as the intensive reclamation of cropland, increased construction land, and landscape fragmentation. We suggest that the Hani should implement scientific land planning and management policies to protect the local ecosystem and realize the sustainable development of traditional ethnic Hani villages.

## 1. Introduction

Traditional ethnic villages are natural or administrative villages with relatively high proportion of ethnic minority population, complete production and living functions, and obvious ethnic minority cultural characteristics. Traditional ethnic villages embody the diversity of civilization and are effective carriers for the inheritance of ethnic culture. With unique natural ecological landscape, they provide effective resources for regional sustainable development and have attracted extensive attention of human beings. The Hani people are mainly distributed in Yunnan Province, China, and are an ancient ethnic minority in China with a long history and unique culture [1]. During mankind’s long history of interactions with nature [2], the Hani people have formed traditional ethnic villages, which reflect their ethnic characteristics, historical culture, farming civilization, and folk customs. For the Hani people, these are important resources that can accelerate the development of the Hani area and have a marked impact on the surrounding environment. However, the rapid development of the economy and urbanization has meant that traditional ethnic Hani villages face destruction in recent years [3], affecting the stability of the surrounding land, landscape patterns, and ecosystems, and ultimately determining whether the Hani traditional ethnic villages can sustainably develop. Therefore, it has become important to evaluate and protect the sustainable development of the Hani traditional ethnic villages. Their spatial distribution characteristics [4–6] and traditional knowledge [7–9] have been extensively studied, though the evaluation and prediction of sustainable development have not been comprehensively analyzed.

Sustainability assessments provide quantitative results and theoretical references for resource utilization, environmental planning, and policy decision-making [10]. Hacking and Guthrie argued that to make sustainability assessments, three categories must be assessed: the direct and indirect environmental, social and economic impacts [10]. Land use change is one of the most important areas of global environmental research [11, 12]; therefore, it is widely used as an environmental pillar in sustainability assessments. Turner integrated land use/cover change into a coupled model of human and environmental systems to assess the characteristics of sustainable land systems [13]. Ecosystem services value (ESV) is closely related to current and future human well-being, and the evaluation of ecosystem services provides a means of planning and managing ecological systems [14]. Matthias incorporated ecosystem services into a framework of sustainability assessment [15], and Daily also proposed to consider ecosystem services in land use decisions [16]. At present, sustainability assessment methods mainly include the use of indicators and targets [17], modeling [18], and multiple-criteria decision analysis [19]. Enormous progress has been made in assessing sustainability over the past 20 years, particularly in the use of indicators and dynamic models. In recent years, the Minimal Cumulative Resistance (MCR) model proposed by Knappen, a Dutch ecologist, has been widely used to assess sustainability [20]. The model describes the resistance that species need to overcome to move from the "source" across the heterogeneous landscape. Zhao proposed that resistance can reduce the impact of human disturbance on the surrounding environment, and a place with low minimum cumulative resistance is suitable for regional expansion and sustainable development [21]. Xue proposed that the value of ecosystem services is an important indicator to assess sustainability, and the regulating services provided by ecosystems can be used as a factor to construct an MCR model [22]. According to Bartlett’s research, population and gross domestic product (GDP) are core indicators that represent human activities and can be chosen to measure human disturbance [23]. Li constructed a sustainable development model of agro-pastoral ecotone based on topography, land use, population, and GDP [24]. Kuang took natural, social, economic, and policy as resistance evaluation factors and used the MCR model to calculate the resistance of expansion for ecological protection land and construction land, to judge the suitability of land expansion [25]. Xiao used the minimum cumulative resistance (MCR) model to evaluate the sustainability of traditional villages in Qiannan Prefecture, China [26]. Great progress has been made in assessing the sustainability of cities or a whole region, but not enough attention has been paid to villages. In this paper, the MCR model was used to integrate ecological factors and human factors, including land use, landscape pattern, ESV, topography, GDP, and population, to construct the resistance surface of the development of Hani traditional villages and evaluate their sustainability, compensating for the lack of assessment of village sustainability and the socio-economic focus in sustainability assessment indicators.

In this paper, typical traditional ethnic Hani villages were selected to analyze their impact in the Hani area on sustainable development, and the MCR model was used to integrate dynamics of land use, landscape pattern, ESV, GDP, and population in a sustainability assessment. This study aims to:(1) Analyze changes in sustainability indicators within 2 km and 4 km buffer zones around traditional ethnic villages; (2) Assess the sustainability of traditional ethnic Hani villages and their impact in typical Hani areas in China from 1995–2020. This paper revealed the spatial and temporal dynamics of sustainability in the Hani area and traditional ethnic villages analyzed the causes for changes in sustainability, filled in the deficiencies of the current evaluation of sustainability in the Hani area, and provided suggestions for land use management planning and sustainable development.

## 2. Materials and methods

### 2.1 Study area

**Fig 1:**
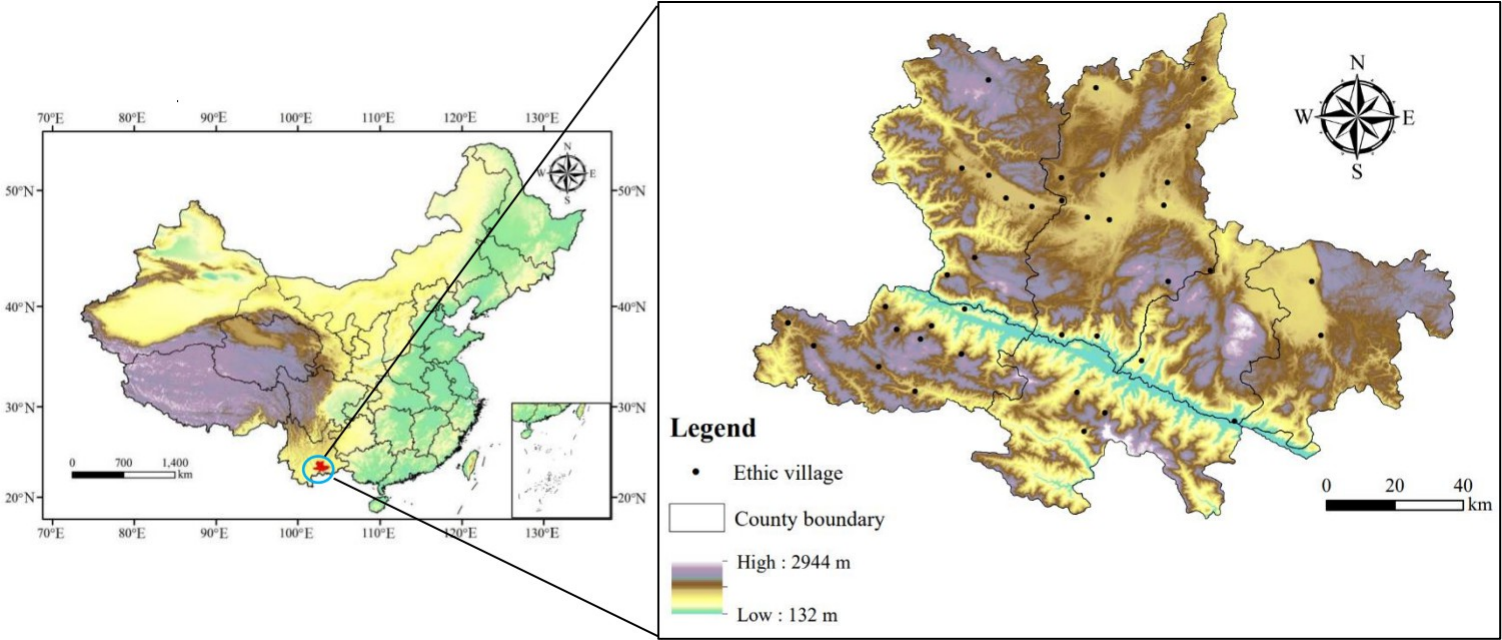
The location of the study area and the traditional ethnic villages.

The Hani people are a cross-border ethnic minority that are also known as the Akha in Southeast Asia (principally in northern Thailand). The Hani mainly live in the Honghe Hani and Yi Autonomous Prefecture of Yunnan Province (hereafter referred to as the “Honghe Prefecture”), China, primarily engage in agriculture, and are skilled in growing tea [27]. We used the Honghe Prefecture, which is located between 22゜26′ N and 24゜45′N and between 101゜47′ and 104゜16′E as a case study. The study area covers six local counties, autonomous counties, or cities in Honghe Prefecture, with a total area of 14,831.32 km^2^. In the study area, the topography is high in the northwest and low in the southeast. It is divided into four parts: mountain range, karst plateau, basin (Bazi), and valley. The Red River Rift Valley divides the territory into two parts: the south is the Ailao Mountain remnant, and the north is the karst plateau with a prominent karst landform (Fig 1). The study area has a subtropical monsoon climate with wet and dry seasons, where the average annual temperature is 16.3℃ and the average rainfall is 2026.5 mm.

As of 2019, the resident population of the study area was 4.78 million, and the urbanization rate reached 34.6%. From 1995 to 2020, the proportion of the Hani ethnic group in the population steadily increased from 16.61% to 19.91%. The Hani people are mainly engaged in agriculture and have a benign agricultural ecosystem of a "forest-village-terraced farms-water system," a diversified mountain landscape, rich rice seed banks, and have developed unique traditional knowledge over time [1]. This paper selected 39 representative traditional ethnic Hani villages located at different altitudes and slopes in the region for the sustainability assessment.

### 2.2 Data source

The Landsat remote sensing data in 1995, 2000, 2005, 2010, 2015 and 2020 were obtained from Resources and Environment Science Data Center Data Registration and Publication System, Chinese Academy of Sciences [28], and combined with research area 1: 250,000 topographic maps, soil and vegetation thematic maps to analyze. The digital elevation model (DEM) data (30 m × 30 m resolution) was obtained from the National Earth System Science Data Sharing Platform [29]. The remote sensing images were preprocessed based on ENVI 4.0 software. Then, according to the supervised classification model and integrated with the regional land use characteristics, the land use types were classified into six categories, mainly including cropland, forest land, grassland, water body, construction land and unused land with accuracy of 85%. Gross domestic product (GDP), food production and population data were sourced from Yunnan Statistical Yearbook and Honghe Prefecture Yearbook in 2021 published by the National Bureau of Statistics [30].

In this paper, the traditional village of data was obtained from the global change science research data publishing system [31], the coordinates of villages were extracted according to three batches of Chinese traditional village directories (2555) successively published by the Ministry of Housing and Urban-Rural Development, the Ministry of Culture and the Ministry of Finance in December 2012, August 2013 and November 2014. Traditional ethnic villages within 2 km were screened and merged according to the distance between villages. Finally, 39 villages with ethnic characteristics of the Hani were taken as the research object of this study. The 39 ethnic villages are distributed in 6 counties and cities of the prefecture, mainly in the northwest of Honghe Prefecture, with the largest number of ethnic villages in Jianshui County, Honghe County and Shiping County, and the least in Gejiu City, Mengzi County and Yuanyang County.

### 2.3 land use change and landscape pattern

#### 2.3.1 Geo-information Tupu change analysis based on GIS

Geo-information Tupu is a classical method to study the transfer direction and quantity change among land use types, which reveals the evolution process of land use pattern [32]. Land use change can be expressed by Geo-information Tupu, which visualizes the mutual transformation of land use types at two time points [33]. In a closed system, from T1 to T2, the transformation of land use types is a mutual process, that is, the increase of one land use type in the system will inevitably lead to the decrease of another land use type.

In order to establish the Tupu of land use change in Hani inhabited areas, ArcGIS 10.4 software was used to make statistics and superposition analysis on the raster data of two periods of land use according to the land use type maps of 1995 and 2020, and obtained the Tupu of land use transfer in 2-km and 4-km buffer zones and Hani area from 1995 to 2020 [34–36]. The formula is as follows:

$$T=Y_{1}\times 10^{n‐1}+Y_{2}\times 10^{n‐2}+\cdots +Y_{n}\times 10^{n‐n}$$ (1)

where, *T* is the Tupu unit code value representing the characteristics of Tupu pattern in the research stage; *Y*_*n*_ is the representation of Tupu unit code value of land use in a given year; *n* is the number of land use types. Tupu codes refer to transitional types of land use change. For example, if the Tupu code is AB, the land use unit code value represented in the previous stage is A, the land use unit code value represented in the next stage is B, and the land use type is converted from A to B.

#### 2.3.2. Landscape pattern analysis based on different buffer zone gradients

Variation of landscape index reflects the landscape pattern change. This paper quantifies landscape pattern by landscape index. To calculating landscape index, Fragstats 4.2 software [37] was used to calculate the landscape indices. A 2 km buffer and a 4 km buffer were made for all ethnic village points in ARCGIS, and land use data for each year were extracted using two types of different scale buffers and imported into Fragstats 4.2 software for index calculation at different distance levels. In this study, four landscape indices were chosen to describe the landscape pattern: Shannon’s diversity index (SHDI) [38], perimeter-area fractal dimension (PAFRAC) [39], landscape shape index (LSI) [40] and contagion index (CONTAG) [41].

### 2.4 Buffer establishment

The impact of traditional villages on the surrounding land can be measured by establishing a buffer zone [42]. In order to further explore the impact of ethnic villages on various indicators of sustainable development, according to the area and precision of the study area, we set up two buffer zones every 2km with ethnic villages as the center using the MultipleRingBuffer tool and r as the radius (r1 = 2 km, r2 = 4 km) in ArcGIS10.4 software, calculated and compared the values of landscape index, ESV and resistance in the buffer zone from 1995 to 2020 [43–45].

### 2.5 Calculating ecosystem service values (ESV)

According to the related researches on ecosystem service value, the value equivalent conversion method was used to determine the ecosystem service value [46–48]. Net profit of ecosystem production was taken as the production value provided by the ecosystem, The net profit of grain production per unit area of cropland ecosystem in 2015 was used as a standard equivalent factor of ecosystem service value. According to the factor table of ecosystem service value equivalent per unit area and the revised value equivalent, the ecosystem service value equivalent of each land use type in Hani area was obtained. The calculation formula of ecosystem service value in Hani area is:

$$ESV=\sum_{i=1}^{n}S_{i}\times V_{c,i}$$ (2)

ESV is the total ecosystem service value; *S*_*i*_ is the area of the *i*th land use type. *V*_*c*,*i*_ are equivalent value of ecosystem services of the *i*th land use type.

According to the Jenks natural breaks classification method in ArcGIS 10.4, the calculation results of ESV are divided into five grades: I, II, III, IV and V.

### 2.6 Sustainability assessment

This study used the MCR model to assess the sustainability of traditional ethic villages. The formula is as follows [49]:

$$MCR=fmin\sum_{j=n}^{i=m}\left(D_{ij}R_{i}\right)$$ (3)

where *f* is a positive correlation function which represents the relative accessibility of the path from the source patch to a certain point in space [50]; *D*_*ij*_ is the spatial distance of landscape base surface *i* traversed by a species from source patch *j* to a point in space [51]; and *R*_*i*_ represents the resistance e of the resistance factor i to the source [52].

The formula for *R*_*i*_ is as follows:

$$R_{i}=\sum_{x=1}^{x=k}\left(W_{x}\times F_{xi}\right)$$ (4)

where *x* represents a factor in the model and *k* represents the number of factors. *F*_*xi*_ is the resistance coefficient of grid cell *i*. The resistance coefficients of the MCR model include intrinsic and external attributes. The internal attributes include land use type, landscape indices, ESV and topography. The external attributes include economy and population. We mark them as "+" depending on the direction of propagation. Ecological resistance represents the obstacle of the ecosystem to human activities, marked as "-". *W*_*x*_ is the weight of each indicator. The raster data of each factor were calculated according to "Technical Guidelines for Environmental Impact Assessment: Ecological Impacts" [53], and *R*_*i*_ was calculated by the raster calculator tool in ArcGIS 10.4. The calculation results and attributes of *F*_*xi*_ are shown in Table 1.

**Table 1:** Classification standards of resistance to development of ethnic villages.

	Rule	Factors	W_x_	F_x_	A	Criteria				
Ecological Resistance	Land use resistance 0.193	Land use type	0.193	F1	-	Construction land	Unused land	Cropland and Waterbody	Grassland	Forest
	Ecosystem service value resistance 0.182	Carbon storage/100 million yuan·hm^-2^	0.059	F2	-	<400	400–800	800–1200	1200–1600	>1600
		Water conservation/100 million yuan·hm^-2^	0.061	F3	-	<1000	1000–1500	1500–2000	2000–2500	>2500
		Soil retention/100 million yuan·hm^-2^	0.062	F4	-	<400	400–800	800–1200	1200–1600	>1600
	Landscape pattern resistance 0.175	SHDI	0.045	F5	-	<0.6	0.6–0.9	0.9–1.2	1.2–1.5	>1.5
		CONTAG	0.044	F6	-	<10	10–20	20–30	30–40	>40
		PD	0.043	F7	-	<0.2	0.2–0.4	0.4–0.6	0.6–0.8	>0.8
		LSI	0.043	F8	-	<1	1.1–1.3	1.3–1.5	1.5–1.7	>1.7
	Topography resistance 0.219	Elevation /m	0.075	F9	-	700–900	900–1100	1100–1300	1300–1500	>1500
		Slope/°	0.073	F10	-	<5	5–10	10–15	15–20	>20
		Topography	0.071	F11	-	plain	hill	basin	valley	mountain
Human Resistance	Social and economic impacts 0.231	GDP density/yuan·km^-2^	0.125	F12	+	<3000	3000–10000	10001–17000	17001–24000	>24000
		Population density/person· km^-2^	0.106	F13	+	<100	100–200	200–300	300–400	>400
		Resistance classification				I	II	III	IV	V
						Lowest	Low	Middle	High	Highest
		Evaluation				10	20	30	40	50

A: The spread direction of F_x_; SHDI: Shannon’s diversity index; SHEI: Shannon′s Evenness Index; LSI: Landscape shape index; CONTAG: Contagion index.

Human disturbance has a variety of expansion routes to expand, and the MCR model was used to calculate the optimal path to expand to any surrounding patch. Then, used the minimum cost distance method to calculate the cumulative resistance of each pathway in ArcGIS 10.4. The higher the resistance value, the more difficult it is for human disturbance to form patches, and the higher the level of sustainability.

The resistance values were classified based on the attributes and the results of the raster calculations. The obtained results were divided into five ranges and visualized in ArcGIS10.4: 0–20%, 20–40%, 40–60%, 60–80%, and 80–100%. Finally, the sustainable zone classification of the study area was obtained and classified into five classes I, II, III, IV and V according to the range of resistance values.

## 3. Results

### 3.1 Land use change based on geo-information Tupu

**Fig 2:**
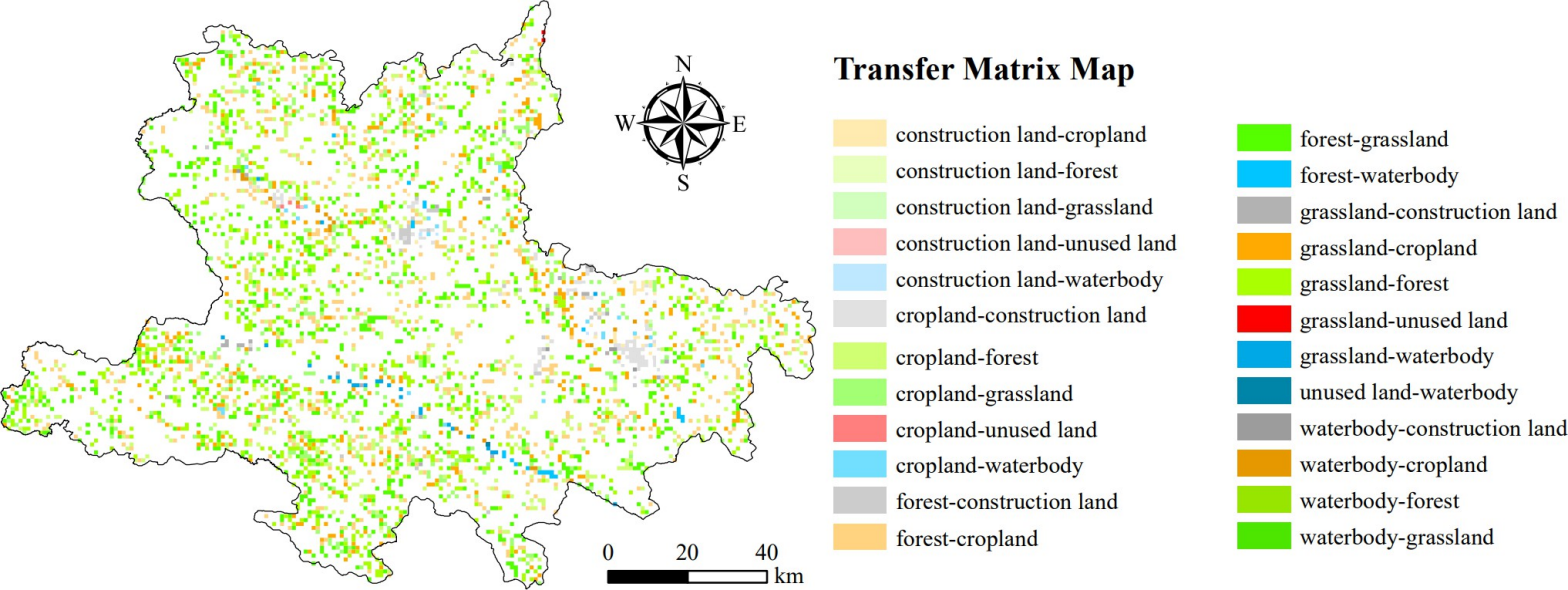
Change of land use type in Hani area from 1995 to 2020.

The temporal and spatial changes in land use types in the Hani area from 1995 to 2020 were significant (Fig 2). The area of construction land and waterbody significantly increased during the research period. Construction land had the biggest proportion of increase, from 7680.00 ha in 1995 to 22,157.15 ha in 2020, increasing by 188.50%. The second largest proportional growth in land use over time is in water bodies, which increased by 47.31%, from 8,964.37 ha in 1995 to 13,205.3 ha in 2020.

Using geo-information Tupu to analyze the land use change in the Hani area from 1995 to 2020, it was found that in the past 25 years, the main type of land use change was the transformation from forest land to grassland, with a total area of 868.62 ha, showing a spatial dispersion. However, among all land use conversion types from 1995 to 2020, construction land converted from woodland and grassland was spatially concentrated, primarily in the northeast and middle of the Hani area.

In addition, based on two buffer gradients of 2 km and 4 km, the transformation of land use types near the traditional ethnic Hani villages from 1995 to 2020 was analyzed by geo-information Tupu. During the study period, there was little difference in land use type transfer between the 2 km and 4 km buffer zones around ethnic villages. In the 2 km buffer zone and the 4 km buffer zone, the transfer of cropland and forest land were the most obvious, accounting for 33.48% and 35.44% of the total transfer area, respectively. The main sources of the transfer of farmland were forest land and grassland, while the main sources of the transfer of forest land were construction land and grassland.

### 3.2 Landscape pattern change around ethnic villages

**Fig 3:**
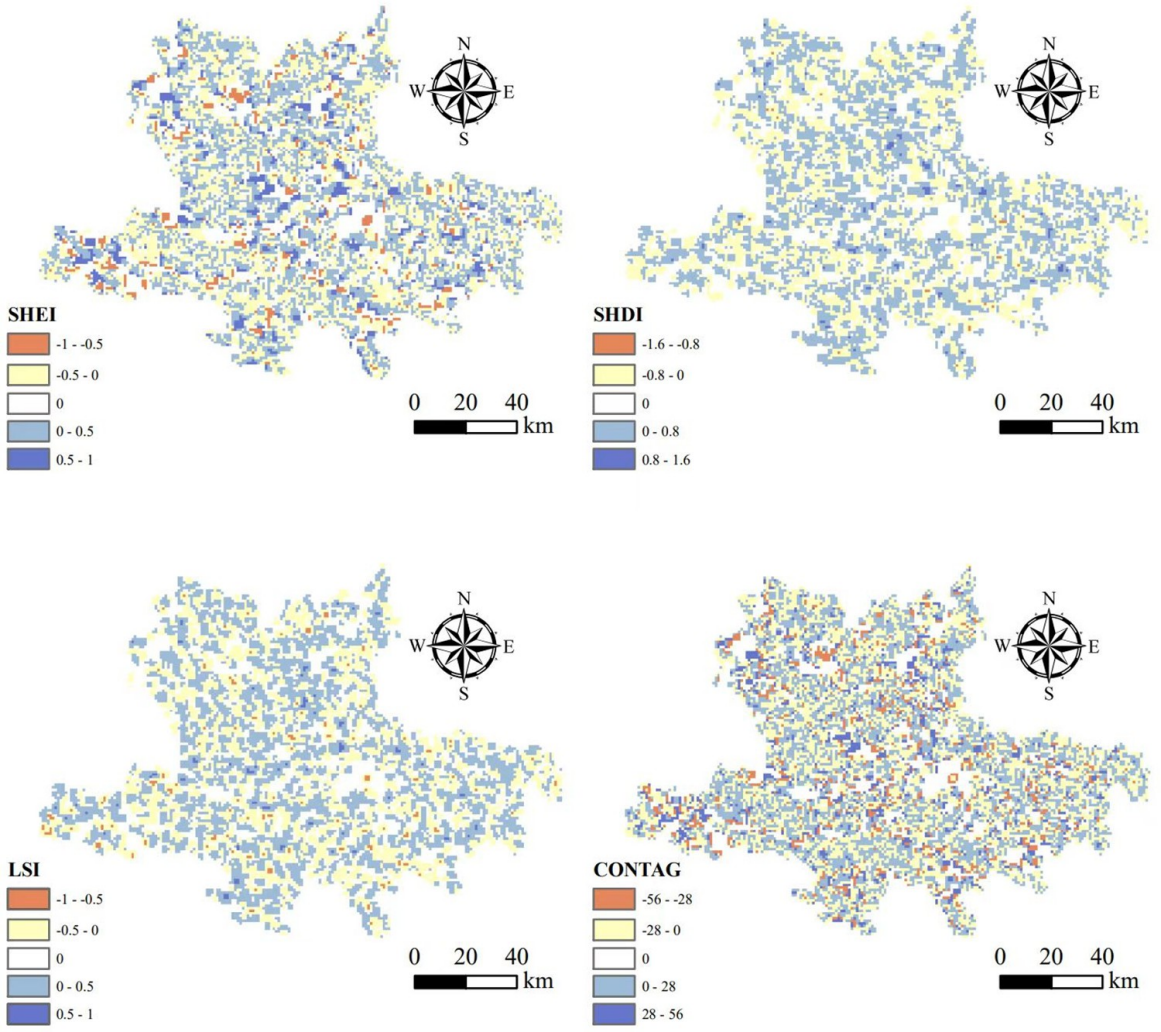
Spatial change of landscape index in Hani area between 1990 and 2020.

From 1995 to 2020, the overall landscape of the Hani area tended to be fragmented and complex (Fig 3). Both the SHDI index and the SHEI index showed an increasing trend year by year, indicating that the patch types tended to be complex and the distribution tended to be balanced. The land use pattern became richer, and the degree of landscape fragmentation increased. The spatial heterogeneity of the two indexes was obvious, and the distribution was uniform in each grading range, which indicated that the land use and the degree of fragmentation in the study area were different. The LSI index increased during the study period, indicating more complex and irregular patch shapes. Spatially, the LSI was evenly distributed, and the main range was -0.5–0.5, indicating that the shape of the landscape was relatively regular. The CONTAG index gradually decreased, indicating that the dominant patches throughout the region are better connected. The distribution of CONTAG index in each area was uniform, indicating that the degree of plaque aggregation in the study area was different.

After calculating the average values of SHDI, SHEI, LSI, and CONTAG indexes for the 2 km and 4 km buffer zones around traditional ethnic villages (Table 2), from 1995–2020, the SHDI, SHEI and LSI indexes for the 2km buffer zone were significantly lower than for the 4km buffer zone, while the opposite was true for the CONTAG index. The overall difference was significant, and the gap between the two gradients remained stable. The results demonstrated that from 1995–2020, the diversity of landscape types near ethnic villages was low, dominated by dominant patches, with regular shapes, low fragmentation, high inter-patch connectivity, and strong extension trends.

**Table 2:** Changes in landscape index in two buffer zones from 1995 to 2020.

Year	Study range	SHDI	SHEI	LSI	CONTAG
1995	2km	0.1662	0.0928	2.7844	92.5362
	4km	0.4605	0.2570	5.6870	80.6801
2000	2km	0.1653	0.0923	2.8282	92.5599
	4km	0.4620	0.2578	5.7389	80.5710
2005	2km	0.1654	0.0923	2.8244	92.5550
	4km	0.4622	0.2579	5.7389	80.5624
2010	2km	0.1654	0.0923	2.8244	92.5550
	4km	0.4624	0.2581	5.7407	80.5504
2015	2km	0.1661	0.0854	2.8282	93.1192
	4km	0.4653	0.2391	5.7796	81.9556
2020	2km	0.1677	0.0936	2.7672	92.4719
	4km	0.4689	0.2410	5.8259	81.8104

SHDI: Shannon’s diversity index; SHEI: Shannon′s Evenness Index; LSI: Landscape shape index; CONTAG: Contagion index.

### 3.3 Spatio-temporal change of ecosystem services value (ESV)

**Fig 4:**
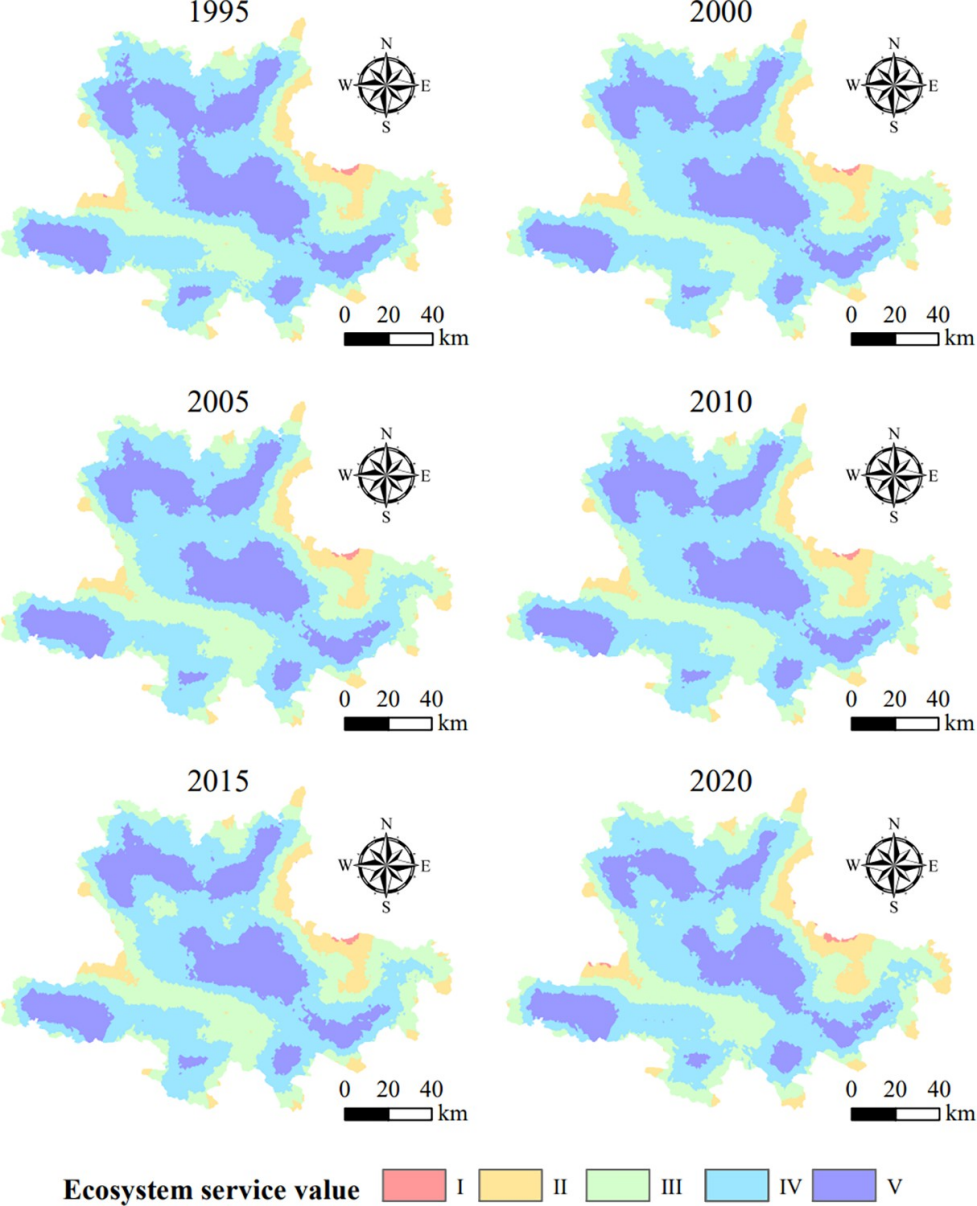
Changes in ecosystem service values.

From 1995 to 2020, the spatial heterogeneity of the value of ecosystem services in the Hani region was evident and high but gradually declined over time. During the study period, the value of ecosystem services was relatively high in the northwest, southwest, and central regions, and low in the east and south. The total value of ecosystem services declined by 2.08% since 1995 and gradually decreased, with a significant decline in the central and northern regions. The decline in ecosystem service values directly affects the well-being of ethnic villages, and ecological problems gradually become more prominent (Fig 4).

In the 2 km and 4 km buffer zones of ethnic villages, the average value of ecosystem services in ethnic villages within 2 km is lower than the average value of ecosystem services within 4 km, and both are lower than the overall level of the whole area. This highlights the high consumption of ecosystem services near ethnic villages. A large amount of land within the 2 km buffer zone of the ethnic village was converted from forest and grassland to cropland during the study period, decreasing the value of ecosystem services (Table 3). From 1995 to 2020, the value of ecosystem services within both the buffer zone and the Hani area decreased each year, indicating that overall ecosystem benefits and functions were in a state of decline.

**Table 3:** Changes in the average ecosystem service values in three gradients.

Study range	1995	2000	2005	2010	2015	2020
2km	8.5532	8.4321	8.4224	8.4203	8.3034	8.3102
4km	8.5811	8.4710	8.4632	8.4612	8.3541	8.3432
Hani Area	8.7424	8.6841	8.6701	8.6731	8.6121	8.5522

### 3.4 Assessment of sustainability

**Fig 5:**
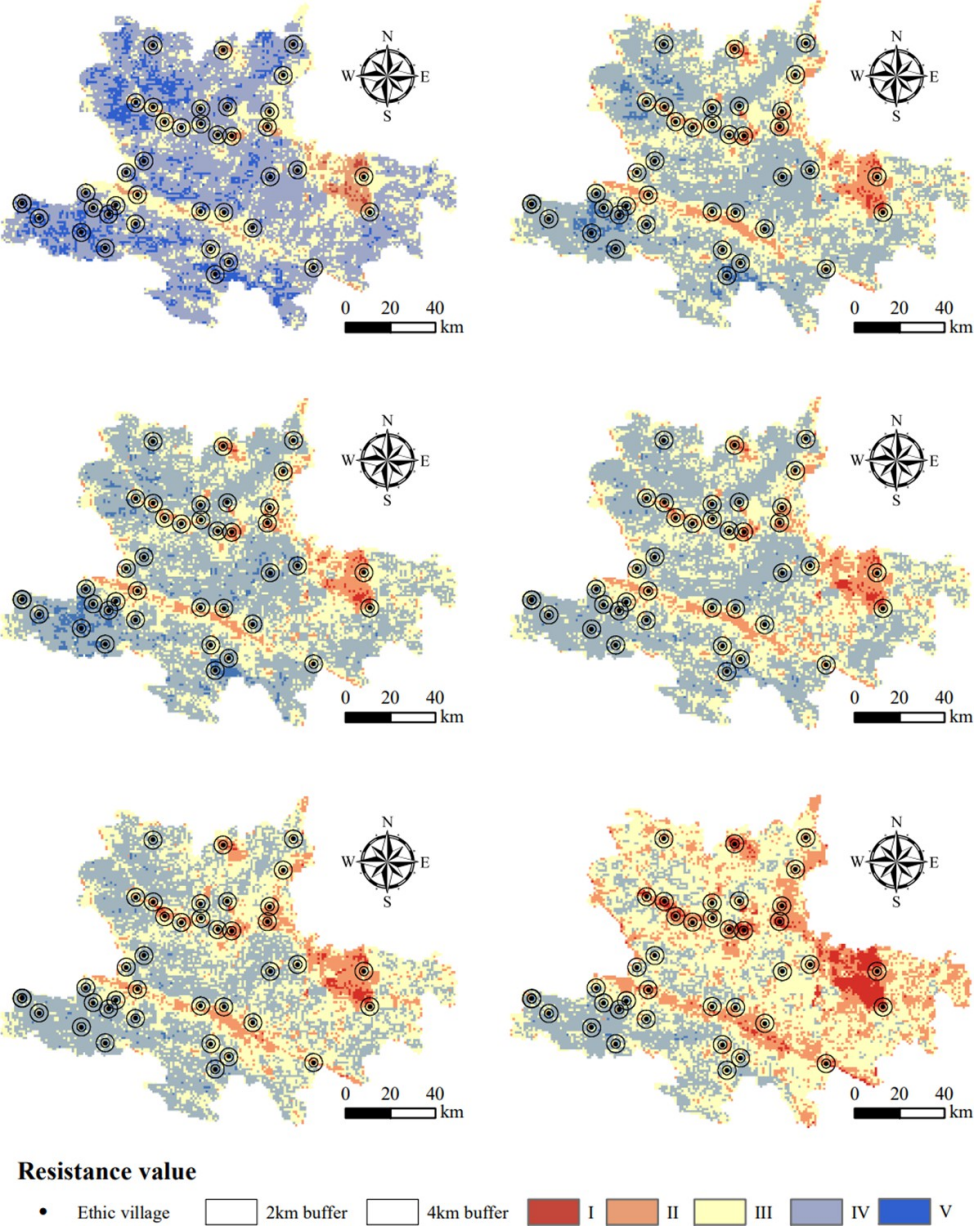
Resistance value of the traditional villages in the Hani area.

According to the results of the sustainability assessment, the level of sustainable development in the Hani area decreased each year from 1995 to 2020, and the spatial heterogeneity also gradually narrowed. Over time, the sustainability level of the low sustainability area in the Northeast further decreased, while the middle and north changed from the high sustainability area to the medium sustainability area, and the spatial heterogeneity of regional development sustainability gradually decreased (Fig 5). In areas where traditional ethnic villages are clustered, the level of sustainability was low and significantly decreased, indicating that ethnic villages had a greater impact on surrounding areas, which reduced landscape stability and landscape ecological security and was not conducive to regional sustainable development.

By analyzing changes in the average resistance values in the 2 km and 4 km buffer zones of ethnic villages, it was found that the average resistance value of the 2 km buffer zone was slightly lower than that of the 4 km buffer zone and lower than that of the Hani area. This indicates that the stability and security of the landscape around ethnic villages were low, and the level of sustainability was low due to human activities. In all three gradients, the level of sustainability gradually decreased over time, and spatially the level of sustainability around ethnic villages in Shiping and Jianshui counties in the northwest was lower than in other ethnic villages, while ethnic villages in Honghe and Luchun counties in the southwest were higher than others (Table 4). This indicates that from 1995 to 2020, the sustainability pattern of traditional ethnic villages in the Hani area gradually became unstable and the impact of human activities gradually expanded.

**Table 4:** Change of the average values of resistance in three gradients.

Study range	1995	2000	2005	2010	2015	2020
2km	20.3420	19.0334	19.4211	18.5320	18.3612	15.7601
4km	20.6013	19.2803	19.6312	18.8223	18.6031	16.3023
Hani Area	21.2941	19.8140	20.1931	19.5034	19.2704	16.9242

## 4. Discussion

### 4.1 Causes for change in sustainability

Land use change, landscape fragmentation, and decreases in ecosystem services are the main reasons for the decline of sustainability in Hani areas. Forest land and grassland were still the main land use types in the Hani area, among which grassland area increased steadily year by year, while construction land also significantly increased. At the same time, landscape fragmentation intensified due to the transformation of some large forest patches into cropland patches, grassland patches, and construction land patches. The values of ecosystem services slightly decreased, and human interference was even worse, which led to a more unsustainable area. Within the 2 km and 4 km buffer zones of ethnic villages in the Hani area, forest land and grassland in the 2 km buffer zone has been largely converted into cropland, resulting in further landscape fragmentation around villages. This led to a reduction in the value of ecosystem services provided by forest land and could lead to more unstable landscape security and lower sustainable development ability. For a long time, the Hani people used terraced fields as the main mode of cultivation due to special geographical conditions and traffic inconveniences [54]. Hani’s terraced fields provide the basic conditions for the survival and development of Hani people and are a reasonable means of communication with the environment formed by Hani people for a long time. However, due to economic development and population growth, the problem of more people and less land is becoming more and more acute. In the Hani inhabited areas, the Hani people develop the forest land near the ethnic village into cropland, and the planting of Hani terraces has reached the limit, leading to the intensification of landscape fragmentation and the reduction of ecosystem services provided by the forest land. The reduction in ecosystem services leads to a decrease in ecosystem regulation, which could lead to additional ecological problems. The plight of more people and less land has also brought about other ecological problems. Local villagers have increased their income by increasing yields and changing their land use structure; one practice is to clear the forest undergrowth to plant higher-yielding grass and fruits and use terraces and dry land for planting cash crops, threatening the continued existence of the terraced landscape. The planting of grass and fruits in the forest weakens water storage capacity and reduces ecological resilience, and private digging and indiscriminate logging happen, making environmental protections even more difficult.

The Hani people live in steep and deep mountainous areas with complex and disparate topography, with a convex central part and low sides, a V-shaped development of the terrain, which is not easy to cultivate, and a mild climate with abundant rainfall, so the area is very sensitive to soil erosion, landslides and terrace collapse during the rainy season. Based on the special geography and climate, the Hani people have formed the Hani terrace cultural ecosystem in a long-term practice, which spatially presents a three-dimensional structure of forest-zhai-field, a closed and sustainable human-land system that meets the normal needs of the Hani people. However, the controller of the whole system is the Hani people, so the stability of the system has only relative significance. Compared with other productivity, the productivity of the terraces is extremely low, and the system remains largely closed to the outside social environment, which can only sustain the system itself, which also leads to a relatively low level of sustainability of the villages compared to the flat areas. However, in the traditional culture of the Hani people, the worship of nature and ancestors based on animism is its core content, and "terraced rice has its own soul" has been their creed for thousands of years. They fear nature and believe that all things are spiritual and have equal rights with human beings. As the most important object of labor and source of livelihood for the Hani people, the terraces are an important guarantee for the continuation of the Hani lineage and are sacred and solemn in the eyes of every Hani, and the forest is an important guarantee for the continuation of the terraces, and the Hani worship trees, forming a unique culture of sacred trees, and prohibiting the cutting of every blade of grass and tree specified as sacred forests. The Hani also developed a top-down irrigation system, which not only made full use of water, but also ensured the safety of the terraces. Terraced planting with running water is a good way to fertilize terraced fields. The Hani people have been consciously protecting the forests and terraces for a long time through the township rules and religious spirits, making it possible for the ecological and productive functions of the forests and terraces to continue for thousands of years without destruction, maintaining the harmonious development of people and land. The important question now is how to maintain the stability of the system in the face of the increasing Hani population and changing environmental awareness. For example, since the 1990s, the Hani people have actively responded to tourism development policies and vigorously built public facilities based on the rich cultural background of the Hani people, which has significantly increased the area of construction land and further threatened the sustainable development of ethnic villages. The development strategy of "one lake and two cities" implemented by Shiping and Jianshui counties has transformed a large amount of land around ethnic villages into construction land, which further reduces sustainable development. However, the sustainable approaches pursued by Honghe County and Luchun County, which mainly protect forest land and cropland, are relatively more stable.

### 4.2 Improving sustainability and recommendations

With the development of the economy and urbanization, traditional ethnic villages must maintain stable development in the face of modern technology and population growth. Sustainable development goals require rational land use planning. However, the sustainable development of traditional ethnic villages is restricted by the demands of the Hani people for economic progress and modernization. To achieve sustainable development, rational land management policies and ecological restoration must be prioritized. First, in areas with low sustainable development, the expansion and development of cropland should be prevented to protect forest land. Meanwhile, ecological restoration projects should be performed, such as returning cropland to forests and grasslands, to improve the supply capacity of ecosystem services. Second, while developing tourism, land should be reasonably planned to prevent the excessive expansion of construction land and increase land utilization. Terrace culture is the basis of tourism development in the Hani area, and it is a key concern and representative feature that cannot be ignored. In policy-making, we should not only consider the need for sustainable development but also pay attention to the protection of Hani’s traditional culture. With the loss of the young and middle-aged population of the Hani people, it is a problem that the farming population is aging. Appropriately reducing cropland is conducive to improving the efficiency of land use and avoiding the problem of large swaths of cropland left uncultivated, facilitating the preservation of terraced culture. In addition, construction land should avoid segmenting the landscape and ecosystem, and forest fragmentation around traditional villages can be restored through afforestation. Finally, for regions with sustainable development, small-scale urbanization activities should be allowed to explore additional benefits of terrace farming, such as increasing the development of terrace farming-related industries led by tourism.

Additionally, the traditional ethnic villages within the Hani area reflect the historical process of the formation and evolution of ethnic areas in different periods and regions, as well as the living conditions, cultural characteristics, and interactions between the ethnic group and the environment in traditional Hani villages. Ethnic landscape planning has experience in how to interact harmoniously with the surrounding environment during long-term production. The Hani people invented the "forest—village terraces—water system" four degrees isomorphism of benign agricultural ecosystem [55], which determines their residence under the sacred forest farther uphill, above and beyond the hill irrigation terraces and causes the reasonable spatial structure and respect for the landscape process. The dense forest above the village provides water, timber, fuel and other resources, which is characterized by the sacred forest of the village god. The terraced fields under the village provide the basic conditions for Hani’s survival and development. The village in the middle forms a place for people to live in peace. The forest, farmland and villages form a three-dimensional spatial structure, perfectly reflecting a sophisticated agricultural, forestry and water distribution system, strengthening the connection between ethnic villages and the surrounding landscape, and providing a valuable reference for the site selection, planning and management of ethnic villages. Hani villages are adjacent to forests above and terraces below, which not only ensure the normal life of villagers, but also provide a good ecological environment and defense environment. Many scholars have done a lot of research on the unique ecosystem formed by the Hani people. Jiao et al. proved that the vertical spatial pattern of the Hani terraced landscape is closely related to the local biogeochemical and hydrological cycles, plays an important role in soil and water conservation, and also supports the food and other cycles that constitute the Hani cultural system. Liu et al. proposed that sustainable sociocultural system is the material guarantee for adaptation to climate change, and it is necessary to give full play to the role of Hani tradition in the maintenance of sociocultural system, so that it can actively participate in climate change adaptation actions at the regional level [56, 57]. In the process of long-term interaction, the Hani people have gradually gained a lot of experience, and many studies have provided useful cases. For example, new rice varieties have been developed on the basis of traditional rice varieties. Various activities related to rice planting, such as planting on rice ridges and raising fish/ducks in rice fields, can also increase yield and enhance the stability of agricultural ecosystems [8, 58, 59].

## 5. Conclusions

Traditional ethnic Hani villages are at risk of ecological environment alteration due to rapid modernization and economic development, and it is important to study their sustainability for their conservation and development. This paper evaluated the sustainability of 39 traditional ethnic villages in the Hani area from 1995 to 2020 using the MCR model with land use type, landscape pattern, ecosystem service value (ESV), and resistance value as indicators. The results indicate that the sustainable development pattern of the Hani area gradually tends to be unstable, while the ethnic villages play a negative role in the sustainable development of the Hani area with increasing human disturbance. During the study period, the area of cropland and landscape fragmentation near ethnic villages significantly increased, the value of ecosystem services gradually decreased, and the level of sustainable development was lower than the overall level. The reasons for this are the increase in population in the traditional Hani ethnic villages, as well as the intensive reclamation of farmland, increased construction land, and landscape fragmentation. In summary, this study explores changes in the sustainability of Hani traditional ethnic villages from 1995 to 2020 and the reasons for them, providing a robust sustainability assessment of Hani and a basis for making decisions about the sustainable revitalization of traditional villages.
